# Joint Effect of Non-invasive Central Systolic Blood Pressure and Peripheral Systolic Blood Pressure on Incident Hypertension in a Chinese Community-based Population

**DOI:** 10.1038/s41598-018-21023-7

**Published:** 2018-02-19

**Authors:** Shixuan Wang, Zechen Zhou, Fangfang Fan, Litong Qi, Jia Jia, Pengfei Sun, Yimeng Jiang, Minghao Kou, Dafang Chen, Yan Zhang, Yong Huo

**Affiliations:** 1 Department of Cardiology, Peking University First Hospital, Beijing, 100034 China; 2 Department of Epidemiology and Biostatistics, Peking University Health Science Center, Beijing, 100191 China

## Abstract

Central blood pressure level is not always consistent with peripheral blood pressure level, and especially their joint effect on incident hypertension is not well established. A total of 1607 non-hypertensive subjects from an atherosclerosis cohort in Beijing, China were included. Central systolic blood pressure (cSBP) was obtained using Omron HEM-9000AI machine and peripheral systolic blood pressure (pSBP) was measured using Omron HEM-7117 electronic sphygmomanometer, separately. Hypertension was defined as BP ≥ 140/90 mmHg or self-reported hypertension or taking any antihypertension drugs at the follow-up survey. After a median follow-up of 2.3 years, incident hypertension was 13.1%. Every 1 standard deviation increase of cSBP and pSBP was associated with 1.98 (95%CI: 1.69–2.33) and 2.84 (95%CI: 2.30–3.52) times of incident hypertension after adjustment for confounders. Moreover, hypertension risk in single pSBP ≥ 120 mmHg group, single cSBP ≥ 120 mmHg group, and both pSBP and cSBP ≥ 120 mmHg group was 2.83 (95%CI: 0.98–8.16), 3.28 (95%CI: 1.24–8.70), and 11.47 (95%CI: 4.97–26.46) times higher than both pSBP and cSBP < 120 mmHg group, respectively. The joint effect of cSBP and pSBP is superior to either cSBP or pSBP to predict incident hypertension in a Chinese community-based population. Screening of central blood pressure should be considered in non-hypertensive population for the purpose of primary intervention, especially for subjects with pSBP ≥ 120 mmHg.

## Introduction

Hypertension is a challenging public health problem worldwide for its high incidence and strong correlation with cardiovascular disease, strokes, and renal outcomes^1–3^. Early identification of high risk population is obvious in its importance for primary prevention of hypertension, especially in less developed countries. Conventional measurement of blood pressure relies on brachial cuff sphygmomanometer due to its convenient and non-invasive characteristic – a practice having been introduced into medical practice for over 100 years. However, arterial blood pressure varies continuously when moving from the aorta to the periphery because of pulse wave amplification across the arterial tree^4^.

Central blood pressure, pressure at the aortic root, can now be reliably estimated by a variety of non-invasive validated devices and is highlighted as the “true pressure” exerted on target organs such as the heart, kidney, and brain^5^. Growing evidence suggests that central pressures are more relevant in predicting cardiovascular risks and target organ damage than brachial pressures^6–9^. Moreover, the reduction of central pressures in hypertensive population appears to be more effective in preventing cardiovascular morbidity and mortality^10,11^, indicating that central blood pressure may be a new worthwhile treatment target for hypertension management. However, few studies have accessed the relation of central blood pressure with future hypertension risk. Recent studies suggest that central blood pressure may be a powerful predictor for future hypertension independent of conventional risk factors^12,13^, but more compelling evidence is still needed. In addition, there are no previous trials investigating the joint effect of central blood pressure and peripheral blood pressure on hypertension risk, so whether central blood pressure can provide incremental value over peripheral blood pressure in risk profile for hypertension prevention remains to be elucidated.

To better understand the different role of central and peripheral blood pressure in the prediction of hypertension, we herein designed this prospective study to investigate the impacts of central systolic blood pressure (cSBP) and peripheral systolic blood pressure (pSBP) on incident hypertension separately and jointly in a Chinese community-based population without hypertension at baseline.

## Results

Among the 1,607 subjects enrolled at baseline, 211 (13.1%) subjects were found to be hypertensive after 2.3-year follow-up at 2014. Table 1 shows the baseline characteristics of study population stratified by whether had hypertension at follow-up survey. For total population, participants were 54.18 ± 7.48 years old and 31.9% were males. Dyslipidemia presented in 65.7% of all participants, whereas 14.9% had diabetes mellitus and 5.4% had cardiovascular disease. Baseline pSBP for subjects who remained normative and who developed hypertension were 121.89 ± 9.77 mmHg and 130.15 ± 7.03 mmHg respectively, and 124.14 ± 13.54 mmHg versus 134.13 ± 13.69 mmHg for cSBP. Subjects who developed hypertension at follow-up visit tended to be older, and had higher male proportion, higher BMIs at baseline compared with subjects who remained normotensive. Prevalence of dyslipidemia, diabetes mellitus, cardiovascular disease and lipid-lowering medication was also higher in subjects who developed hypertension at follow-up visit.

**Table 1:** Baseline characteristics of the study population, overall and according to blood pressure status at follow-up.

Variable	Total study population	Normotensive at follow-up	Hypertensive at follow-up	*P*
	*n* = 1,607	*n* = 1,396	*n* = 211	
Age, mean ± SD, y	54.18 ± 7.48	53.83 ± 7.24	56.47 ± 8.60	< 0.001
Men, n (%)	513 (31.9)	431 (30.9)	82 (38.9)	0.021
BMI, mean ± SD, kg/m^2^	25.23 ± 3.25	25.05 ± 3.20	26.46 ± 3.30	< 0.001
pSBP, mean ± SD, mmHg	122.97 ± 9.86	121.89 ± 9.77	130.15 ± 7.03	< 0.001
pDBP, mean ± SD, mmHg	71.42 ± 7.64	71.00 ± 7.54	74.16 ± 7.76	< 0.001
cSBP, mean ± SD, mmHg	125.45 ± 13.97	124.14 ± 13.54	134.13 ± 13.69	< 0.001
eGFR, mean ± SD, mL/min per 1.73 m^2^	97.48 ± 11.17	97.82 ± 10.96	95.22 ± 12.29	0.002
Current smoking, n (%)	320 (19.9)	272 (19.5)	48 (22.7)	0.268
Current drinking, n (%)	369 (23.0)	313 (22.4)	56 (26.5)	0.185
Dyslipidemia, n (%)	1056 (65.7)	899 (64.4)	157 (74.4)	0.004
Diabetes mellitus, n (%)	2490 (14.9)	192 (13.8)	47 (22.3)	0.001
Cardiovascular disease, n (%)	87 (5.4)	69 (4.9)	18 (8.5)	0.032
Lipid-lowering medications, n (%)	83 (5.2)	65 (4.7)	18 (8.5)	0.018
Hypoglycemic medications, n (%)	94 (5.8)	77 (5.5)	17 (8.1)	0.144

BMI indicates body mass index; pSBP, peripheral systolic blood pressure; pDBP, peripheral diastolic blood pressure; cSBP, central systolic blood pressure; eGFR, estimated glomerular filtration rate.

**Figure 1:**
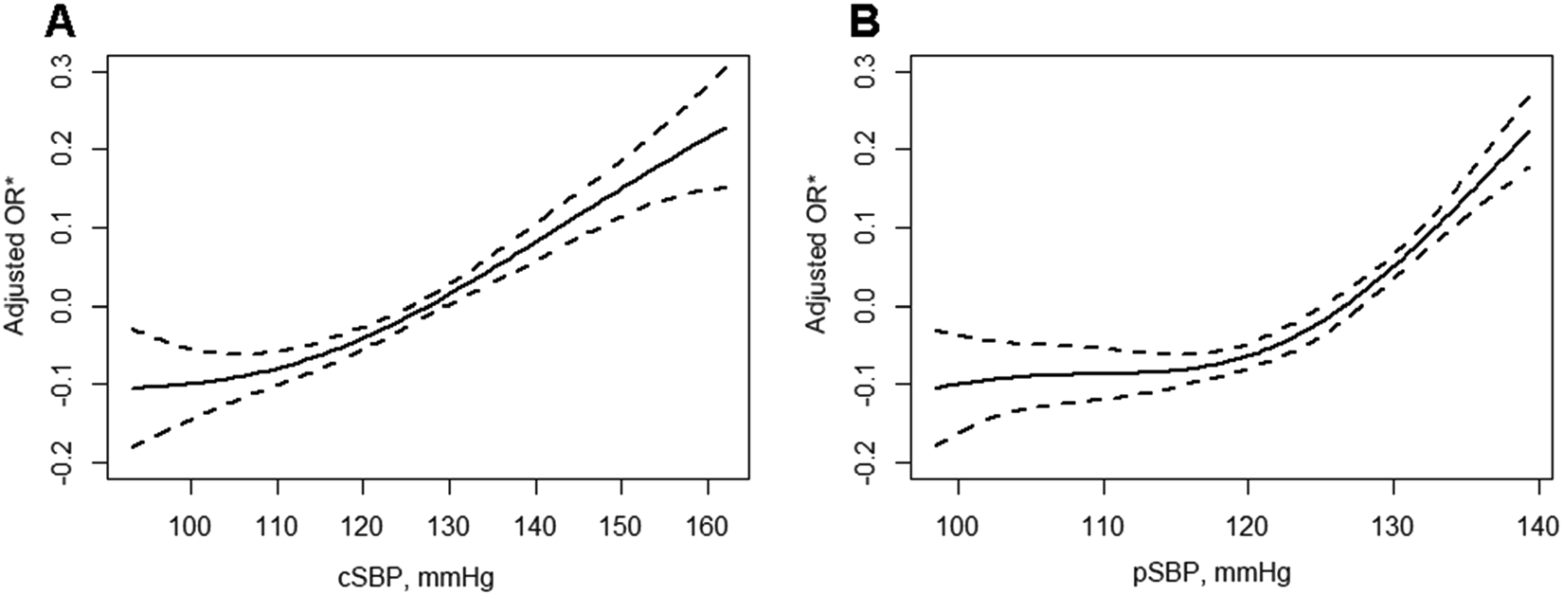
Multivariable adjusted spline curves for relation between cSBP (**A**), pSBP (**B**) and risk of hypertension. *Adjusted for age, sex, body mass index, current smoking and drinking status, dyslipidemia, diabetes mellitus, history of cardiovascular disease, lipid-lowering medications, hypoglycemic medications and baseline eGFR.

In the spline model adjusted for potential confounders, the predictive risk of new-onset hypertension generally increased with rising level of cSBP and pSBP (Fig. 1). A roughly positive linear correlation was observed between cSBP and hypertension risk, whereas a non-linear relationship was observed for pSBP. Of note, the inflection point of 118.33 mmHg for pSBP in the spline curve was very close to the cutoff value of 120 mmHg for prehypertension state in clinical practice.

Table 2 shows the results of multivariate regression analysis for incident hypertension in relation to cSBP and pSBP. Both cSBP and pSBP were associated with hypertension risk independently even after adjusting for confounders. The risk for developing hypertension increased 98% [adjusted OR = 1.98, 95% confidence interval (CI): 1.69–2.33)] for per 1 standard deviation (SD) increase in cSBP and 184% (adjusted OR = 2.84, 95% CI: 2.30–3.52) for per 1 SD increase in pSBP. In addition, compared with <120 mmHg group, the adjusted odds ratios for incident hypertension were 4.73 (95% CI: 3.01–7.46) for cSBP ≥ 120 mmHg group and 4.18 (95% CI: 2.67–6.54) for pSBP ≥ 120 mmHg group respectively.

**Table 2:** Relations of cSBP and pSBP to the risk of incident hypertension.

Variable	*n*	Incidence of hypertension	Crude Model		Multivariate-Adjusted Mode 1*		Multivariate-Adjusted Mode 2^†^	
		*n* (%)	OR (95% CI)	*P*	OR (95% CI)	*P* Value	OR (95% CI)	*P*
**cSBP**								
cSBP (per 1SD)	1607	211 (13.1)	2.04 (1.76–2.38)	<0.001	2.00 (1.71–2.33)	<0.001	1.98 (1.69–2.33)	<0.001
cSBP < 120 mmHg	557	23 (4.1)	1		1		1	
cSBP ≥ 120 mmHg	1050	188 (17.9)	5.06 (3.24–7.91)	<0.001	4.81 (3.07–7.53)	<0.001	4.73 (3.01–7.46)	<0.001
**pSBP**								
pSBP (per 1SD)	1607	211 (13.1)	3.03 (2.48–3.71)	<0.001	2.91 (2.37–3.57)	<0.001	2.84 (2.30–3.52)	<0.001
pSBP < 120 mmHg	585	25 (4.3)	1		1		1	
pSBP ≥ 120 mmHg	1022	186 (18.2)	4.98 (3.24–7.67)	<0.001	4.57 (2.96–7.06)	<0.001	4.18 (2.67–6.54)	<0.001

cSBP indicates central systolic blood pressure; pSBP, peripheral systolic blood pressure.

*Adjusted for age, sex.

^†^Adjusted for age, sex, body mass index, current smoking and drinking status, dyslipidemia, diabetes mellitus, history of cardiovascular disease, lipid-lowering medications, hypoglycemic medications and baseline eGFR.

Joint effect of cSBP and pSBP on hypertension risk based on the four defined groups is shown in Table 3. Subjects in both pSBP and cSBP ≥ 120 mmHg group showed a significantly higher incidence of new-onset hypertension at follow-up survey compared with the other three groups. Compared with both pSBP and cSBP < 120 mmHg group, the risk for hypertension in single pSBP ≥ 120 mmHg group, single cSBP ≥ 120 mmHg group, and both pSBP and cSBP ≥ 120 mmHg group was 2.83 (95% CI: 0.98–8.16), 3.28 (95% CI: 1.24–8.70), and 11.47 (95% CI: 4.97–26.46) times respectively in the multi-adjusted model.

**Table 3:** Relations of the four defined groups to the risk of incident hypertension.

Group (mmHg, mmHg)	*n*	Incidence of hypertension	Crude Model			Multivariate-Adjusted Mode 1*			Multivariate-Adjusted Mode 2^†^		
		*n* (%)	OR (95% CI)	*P*	*P* for trend	OR (95% CI)	*P* Value	*P* for trend	OR (95% CI)	*P*	*P* for trend
G1 (pSBP < 120, cSBP < 120)	315	6 (1.9)	1		<0.001	1		<0.001	1		<0.001
G2 (pSBP ≥ 120, cSBP < 120)	242	17 (7.0)	3.89 (1.51–10.03)	0.005		2.89 (1.10–7.61)	0.032		2.83 (0.98–8.16)	0.054	
G3 (pSBP < 120, cSBP ≥ 120)	270	19 (7.0)	3.90 (1.53–9.91)	0.004		3.58 (1.40–9.19)	0.008		3.28 (1.24–8.70)	0.017	
G4 (pSBP ≥ 120, cSBP ≥ 120)	780	169 (21.7)	14.25 (6.24–32.53)	<0.001		13.15 (5.74–30.12)	<0.001		11.47 (4.97–26.46)	<0.001	

^*^Adjusted for age, sex.

^†^Adjusted for age, sex, BMI, current smoking and drinking status, dyslipidemia, diabetes mellitus, history of cardiovascular disease, lipid-lowering medications, hypoglycemic medications and baseline eGFR.

## Discussion

The present study shows that both cSBP and pSBP were independently associated with future hypertension incidence, while different patterns were observed in the spline model. More importantly, combined detection of cSBP and pSBP would considerably improve the identification of people with elevated hypertension risk, thereby lending support to the hypothesis that central blood pressure measurement may be an attractive approach for primary prevention of hypertension. Our study also provides additional support for the recent perspective that central blood pressure not only served as an endpoint, but could also be a predictor of future cardiovascular risk in clinical practice^14^.

The value of brachial artery pressure in the epidemiology of hypertension has been firmly established, embodied in guidelines for hypertension management. As non-invasive devices introduced to medical practice, greater emphasis has been placed on central pressures recently for they can more exactly reflect the pressures loading on target organs such as the central nervous system, the heart and the kidney. The most influential study drawing attention to the potential superiority of central blood pressure over brachial blood pressure was the CAFÉ substudy of the ASCOT trial^11,15^. In this substudy, differential effects on central pressures were reported for different blood pressure lowering drugs despite similar effects on brachial pressures, which might explain the “beyond blood pressure benefits” of cardiovascular stroke and survival in the calcium channel blocker (amlodipine) group compared with the beta-blocker (atenolol) group. Subsequent studies pointed out that central pressures were more closely correlated with intermediate measures of cardiovascular risk (i.e. carotid intima media thickness, left ventricular mass) than corresponding brachial blood pressures^6,9,16,17^. Further, some longitudinal observations demonstrated that central pressures were more predictive of cardiovascular events and mortality than brachial pressures^6,8,9^. However, much less is known for central blood pressure in the prediction of hypertension risk. In this context, the present work would contribute to the knowledge of the role of cSBP as potential complementary to brachial blood pressure in hypertension prevention and management.

Our findings of an association between cSBP and an increased risk of hypertension are consistent with previous studies. In a recent study of middle-aged Japanese men, Tomiyama suggested that central aortic systolic blood pressure could predict future hypertension more reliably than brachial-ankle pulse wave velocity independent of traditional risk factors^12^. Another prospective observational study conducted in a cohort of young to middle-aged participants with stage 1 hypertension reported that central blood pressure was an independent predictor of future antihypertensive treatment^13^. However, the combined effect of cSBP and pSBP on incident hypertension has not been described in prior studies. Data from our cohort suggest that central blood pressure could provide increment predictive value in addition to brachial blood pressure for future hypertension risk as seen by the significantly higher hypertension incidence in the both pSBP and cSBP ≥ 120 mmHg group.

As to our knowledge, central blood pressure is invariably lower than corresponding brachial blood pressure based on invasive catheter measurement. Contrary to this fact, in our study, the average cSBP of total population was higher than corresponding pSBP and 270 (16.8%) subjects were assigned to the pSBP < 120 mmHg and cSBP ≥ 120 mmHg group. This can be explained by the cuff errors of brachial blood pressure measurement and the technological approach of Omron device. The Omron device records the radial pulse waveform and obtains the second peak of the peripheral systolic pressure (SBP2) by calibrating the radial waveform with brachial blood pressure^18^. Because cuff measured brachial blood pressure underestimates catheter brachial blood pressure by approximately 18 mmHg^19^, the SBP2 would substantially underestimate cSBP whenever the cuff measured brachial blood pressure was used for calibration. To relieve the influence of the cuff pressure on cSBP estimation and bridge the gap between noninvasive and invasive measurements, the Omron device introduced a regression equation to calculate the non-invasive cSBP with SBP2 asserting into the equation as a major independent variable^18^. As a result, cSBP provided by the Omron device is usually higher than corresponding cuff measured brachial systolic blood pressure under many instances, but is more closely to the invasive cSBP^19^.

This observational study does not provide a causal relation between cSBP and the development of hypertension. However, there are several possible pathophysiological mechanisms supporting an association between cSBP and hypertension risk. Specifically, the pressure wave generated by the left ventricle travels from the high elastic aortic artery to the stiffer periphery and is reflected at bifurcations and caliber disparities^5^. The disparity between central and peripheral blood pressure is mainly determined by the pulse pressure amplification. Therefore, any factors causing increased stiffness of aortic artery and wave reflection would augment central blood pressure independently of brachial blood pressure^20^. Indeed, recent cross-sectional studies have reported a stronger association of central aortic pressure with carotid wall remodeling and increased arterial stiffness than brachial blood pressure, even in normotensive subjects^21–23^. In light of the fact that increased aortic arterial stiffness would proceed hypertension^24–27^, it is reasonable to deduce that central blood pressure as a surrogate maker of arterial stiffness may provide valuable instructions for hypertension prevention^14,24^. Further, in the Anglo-Cardiff Collaborative Trial II Study, McEniery observed that individuals presented with higher central blood pressure had more cardiovascular risk factors or diseases despite similar peripheral blood pressure, and over 70% of individuals with high normal peripheral blood pressure had similar central blood pressure as those with stage 1 hypertension, >30% of men and 10% of women with normal peripheral blood pressure had central blood pressure in common with subjects with stage 1 hypertension^28^. On condition that hypertension shares similar risk factors with cardiovascular disease (i.e. hyperlipidemia, hyperglycemia, obesity)^29–31^, a higher central blood pressure level might indicate a greater potential of progression to hypertension. Results of our study reinforced this hypothesis for a higher odds ratio for incident hypertension was observed in the single cSBP ≥ 120 mmHg group compared with the single pSBP ≥ 120 mmHg group.

Several limitations in this study should be addressed. First, until now, there is no established standard reference value for cSBP. In this study, we defined subgroups with a cut-off value of 120 mmHg for both pSBP and cSBP based on guidelines of peripheral blood pressure and previous works on central blood pressure. This would inevitably cause diminished accuracy for central blood pressure categorization. Besides, technological differences between different validated devices would also lead to differential results in various studies. Second limitation of this study was the heterogonous definition of new-onset hypertension, defined as brachial blood pressure ≥140/90 mmHg or self-reported hypertension or taking any antihypertension drugs at the follow-up survey. In case subjects taking antihypertension drugs for other reason (i.e. cardiovascular disease), we performed a separate multivariate analysis. Exclusion of individuals taking any antihypertensive drugs at follow-up visit did not alter the results (data not shown). Third, although not necessarily a limitation, the correlation between blood pressure indices and incident hypertension did not indicate causal relationships given the nature that this is a longitudinal observational study.

## Methods

### Study population and design

The study population was from a community-based atherosclerosis cohort in Beijing, China, details of which have been published previously^32^. Briefly, from December 2011 to April 2012, residents aged above 40 years in the Gucheng and Pingguoyuan communities of the Shijingshan District were enrolled either by responding to the study recruitment posters or by inviting phone calls if the community health centers had their health medical records. A follow-up survey was conducted among subjects who had gene chip data in the year of 2014, from May to July. Participants fulfilling the following criteria were included in this substudy: (1) completed both the baseline and follow-up visit; (2) had no established hypertension and baseline brachial blood pressure <140/90 mmHg; (3) did not take any antihypertensive drugs at baseline visit; (4) had successful central and peripheral blood pressure measurements. Finally, a total of 1607 eligible subjects were included in our final analysis. All participants gave informed consent and the study was approved by the ethics committee of both Peking University and Peking University First Hospital. The methods were carried out in accordance with relevant guidelines and regulations.

### Data collection

#### Demographic variables

Participants completed a standardized written questionnaire concerning socio-demographic and medical data including age, gender, occupation, education, lifestyle factors, comorbid conditions and medical history. Current smoking was defined as cigarette consumption of one cigarette per day for at least half a year. Current drinking was defined as alcohol intake once per week for at least half a year. Body mass index (BMI) was calculated as weight (kg) divided by the square of height (m).

#### Biochemical variables

Overnight fasting blood samples were drawn for glucose, lipid, serum creatinine and other biochemical measurements. All baseline laboratory variables were tested using the Roche C8000 Automatic Analyzer (Roche Diagnostics, Indianapolis, IN, USA). Chronic Kidney Disease Epidemiology Collaboration (CKD-EPI) equation was used to determine glomerular filtration rate (eGFR) based on serum creatinine^33^. Diabetes mellitus was defined as fast blood glucose ≥7.0 mmol/L, or oral glucose tolerance test (OGTT) ≥11.1 mmol/L, or any self-reported history of diabetes.

#### Blood pressure variables

Measurements of central blood pressure were conducted with an automated device (HEM-9000AI; Omron Healthcare, Kyoto, Japan) using the method of radial artery applanation tonometry^18^. Left radial artery pressure waveforms and right brachial blood pressure were measured simultaneously after the subjects enduring a rest in sitting position for at least 5 min. Then we obtained the first and second peaks of the peripheral systolic pressure (SBP1 and SBP2) by calibrating the radial waveform with brachial systolic blood pressure. Finally, central blood pressure was automatically calculated with a linear equation for SBP2.

Peripheral blood pressure was measured using a validated automatic arm device (HEM-7117; Omron Healthcare, Kyoto, Japan) with appropriately sized cuffs following a standard protocol. With the participant being seated in a quiet room for a 5-minute rest, three blood pressure readings were obtained at intervals of no less than 1 minute. The average of the 3 consecutive readings was recorded as the final pSBP and peripheral diastolic blood pressure (pDBP) value. Simultaneously pulse at rest was obtained. New onset of hypertension was defined as blood pressure ≥140/90 mmHg or self-reported hypertension or taking any antihypertension drugs at the follow up survey.

### Statistical analysis

All statistical analyses were carried out using SPSS 19.0 (SPSS, Chicago, IL, USA) and Empower (R) (www.empowerstats.com, X&Y solutions, Inc. Boston MA). Data were expressed as mean ± standard deviation (SD) for continuous variables and percentages (%) for dichotomous variables. Comparison of continuous variables was performed using an independent *t*-test, and categorical variables were compared using chi-square analysis. Multivariate logistic regression models were used to investigate the independent and joint relations of cSBP and pSBP to new onset of hypertension. Covariates adjusted in the models included age, sex, BMI, current smoking and drinking status, dyslipidemia, diabetes mellitus, history of cardiovascular disease, lipid-lowering medications, hypoglycemic medications and baseline eGFR. The spline curves were also plotted to obtain additional insights into the association between cSBP, pSBP and hypertension risk.

To examine the joint effect of pSBP and cSBP on new onset of hypertension, we defined four subgroups according to their baseline cSBP and pSBP levels. Current hypertension guidelines recommended <120/80 mmHg as normal, and 120–139/80–89 mmHg as prehypertensive for peripheral blood pressure^34,35^, while there was no established standard reference value for cSBP. A recent study proposed <110/80 mmHg as optimal and ≥130/90 mmHg as hypertensive for central blood pressure with a SphygmoCor device^36^, whereas in previous studies cSBP provided by an Omron device was approximately 13 mmHg higher than that of SphygmoCor^19,37^. Considering that people with prehypertensive state are at increased risk of progression to hypertension, we adopted the cut-off value of 120 mmHg for both pSBP and cSBP in defining the four subgroups, as follows:

1. Group 1 (G1): pSBP < 120 mmHg, cSBP < 120 mmHg;
2. Group 2 (G2): pSBP ≥ 120 mmHg, cSBP < 120 mmHg;
3. Group 3 (G3): pSBP < 120 mmHg, cSBP ≥ 120 mmHg;
4. Group 4 (G4): pSBP ≥ 120 mmHg, cSBP ≥ 120 mmHg.

### Data availability

The datasets generated during the current study are available from the corresponding author on reasonable request.

## Conclusion

In conclusion, our findings suggest that the joint effect of cSBP and pSBP is superior to either cSBP or pSBP to predict incident hypertension in a Chinese community-based population without hypertension. Thus, non-invasive central blood pressure screening should be considered in non-hypertensive population as a routine health examination for the purpose of primary prevention of hypertension, especially for people with pSBP ≥ 120 mmHg.
